# Linalool, derived from *Cinnamomum camphora* (L.) Presl leaf extracts, possesses molluscicidal activity against *Oncomelania hupensis* and inhibits infection of *Schistosoma japonicum*

**DOI:** 10.1186/1756-3305-7-407

**Published:** 2014-08-29

**Authors:** Fan Yang, Erping Long, Juhua Wen, Lei Cao, Chengcheng Zhu, Huanxin Hu, Ying Ruan, Kamolnetr Okanurak, Huiling Hu, Xiaoxia Wei, Xiangyun Yang, Chaofan Wang, Limei Zhang, Xiaoying Wang, Pengyu Ji, Huanqin Zheng, Zhongdao Wu, Zhiyue Lv

**Affiliations:** Zhongshan School of Medicine, Sun Yat-sen University, 74 2nd Zhongshan Road, Guangzhou, 510080 China; Key Laboratory for Tropical Diseases Control of Ministry of Education, Sun Yat-sen University, Guangzhou, 510080 China; Department of Physiology, Hunan University of Chinese Medicine, Changsha, 410208 China; Department of Social and Environmental Medicine, Faculty of Tropical Medicine, Mahidol University, Bangkok, 10400 Thailand

**Keywords:** Linalool, *Oncomelania hupensis*, *Schistosoma japonicum*, Molluscicidal activity, Schistosomicidal property, *Cinnamomum camphora*

## Abstract

**Background:**

Schistosomiasis japonicum remains a considerable economic and public health concern in China, the Philippines and Indonesia. Currently available measures to control the unique intermediate host *Oncomelania hupensis* are frequently associated with severe side effects. Previous studies have demonstrated that linalool-rich extracts from various plants exhibited promising biological activities including cytotoxic, anti-microbial and anti-parasitic properties.

**Methods:**

We identified the components of leaf extracts from *Cinnamomum camphora* by gas chromatography coupled to mass spectrometry (GC-MS) and investigated molluscicidal and larvicidal effects of linalool against *O. hupensis* and *Schistosoma japonicium*. The ultrastructural alterations in gills, salivary gland, stomach and hepatopancreas of snails were observed under the light microscope and transmission electron microscope, and lesions to tegument of cercaria were examined under a light microscope and fluorescence microscope. We then evaluated the effects of linalool on skin penetration and migration of schistosomula and adult survival by measurement of worm burden and egg counts in Balb/C mice infected with linalool-treated cercariae.

**Results:**

In the present work, 44 components were identified from the leaf extracts of *C. camphora*, of which linalool was the most abundant constituent. Linalool exhibited the striking molluscicidal and larvicidal effects with LC_50_ = 0.25 mg/L for *O. hupensis* and LC_50_ = 0.07 mg/L for cercaria of *S. japonicium*. After exposure to linalool, damage to the gills and hepatopancreas of the snails, and to the tegument and body-tail joint of cercariae was apparent. In addition, linalool markedly reduced the recovered schistosomulum from mouse skin after challenge infection, and therefore decreased the worm burden in infected animals, but not fecundity of female adults of the parasite.

**Conclusions:**

Our findings indicated that linalool might be a novel chemotherapeutic agent against *S. japonicium* and the snail intermediate host.

## Background

Schistosomiasis japonicum, a parasitic disease caused by *Schistosoma janponcium* that live in snail-infested fresh water, remains one of the most prevalent parasitic infections with 413,000 people suffering from the plague in 7 provinces of China (Hubei, Hunan, Jiangxi, Anhui, Jiangsu, Sichuan and Yunnan) [1]. Endemicity of schistosomiasis japonicum is governed by socioeconomic and behavioral determinants, and the distribution of the snail intermediate host *Oncomelania hupensis*, which mainly spread in the Yangtze River valley and mountains or hills in southwest China. Though *O. hupensis* control by molluscicides is one of the main strategies to reduce the worm transmission in the snail population in infected areas, the synthetic niclosamide (2′, 5-dichloro-4′-nitrosalicylanilide) is the only effective molluscicide recommended by World Health Organization [2]. Studies on the molluscicidal efficacies of natural products of plant origin have been promoted due to the high cost and environmental contamination of synthetic molluscicides, and the possible development of snail resistance [3–5].

Extracts from several species of the *Cinnamomum* have shown to be one of the most promising sources of new bio-efficacy against fungus [6, 7], bacteria [8] and insect pests [9], and compounds for treatment of infectious diseases [10], autoimmune diseases [11] and cancer [12]. There are few reports on molluscicidal activity of leaf extracts from *C. camphora* against *O. hupensis* [13, 14]. The schistosomicidal effect of the extracts form *C. camphora* and the underlying mechanisms that account for their activity and active components, however, remain largely unelucidated. In this study, therefore, we identified the active component of extracts from leaves of *C. camphora* and elevated the molluscicidal effect against *O. hupensis* and the schistosomicidal activity.

## Methods

### Compounds, leaves and experimental animals

Linalool was purchased from Sigma–Aldrich Chemical Co., China. Niclosamide and wild adult negative snails of *O. hupensis* were gifts from Leading Office of Schistosomiasis Control of Hanchuan County, Hubei Province, China, and the positive snails were kindly presented by Prof. Shiping Wang, Department of Parasitology, Xiangya School of Medicine, the Central South University (Changsha, China). The two kinds of snails were confirmed by cercaria shedding test in our laboratory and reared under standard laboratory conditions. The leaves of *C. camphora* were collected in July 2011 from North Campus, Sun Yat-sen University, China. Voucher samples were deposited in Sun Yat-sen University Department of Parasitology collection with a reference number of 2011060018. Eight-week-old Balb/C mice were purchased from the Laboratory Animal Center of Sun Yat-sen University (Guangzhou, China). The mice infected with cercariae were used to determine the inhibition of cercarial penetration and protective effect of linalool against *S. japonicum*. All of the procedures involving animals and their care in this study were approved by the Institutional Animal Care and Use Committee of Sun Yat-sen University in accordance with institutional guidelines for animal experiments.

### SC-CO_2_ extraction and GC-MS analysis

The fresh healthy leaves of *C. camphora* were picked from the sunlit branches of trees. Air-dried leaves (100 g) of the tree were powdered and extracted according to the protocol in previously published articles [15, 16] with minor modifications in a supercritical CO_2_ extraction apparatus (HA121-50-12, Huaan Supercritical Fluid Extraction Ltd., China). The sample was extracted with supercritical fluid CO_2_ at 25 MPa at 45°C for 4 hours and at the desired flow rate 15 L/h.

The components of extraction (0.5 μL), clear and of golden color, were quantified and identified by a QP2010 gas chromatography-mass spectrometry (GC-MS, Shimadzu Corporation, Japan). Injector temperatures were set at 280°C and the initial GC column temperature was held at 40°C for 5 min, then increased to 250°C at a rate of 5°C/min and maintained for 5 min at this final temperature. Helium was used as the carrier gas at 0.8 mL/min. The transfer line temperature was 230°C. Electron impact mass spectra were scanned at 70 ev in the mass range of 40-600 units. Compounds were identified by comparison of their mass spectra with those of NIST05 library data of the GC-MS system.

### Molluscicidal assay

Molluscicidal activities of extracted samples and linalool against *O. hupensis* were evaluated following protocols in previously described studies [17, 18]. Briefly, 30 negative snails per group were placed in a 250-ml wide-necked bottle and maintained for 6 h, 12 h, 18 h, 24 h, 30 h, 36 h, 42 h, 48 h, 54 h, 60 h, 66 h, 72 h, 78 h, 84 h, 90 h or 96 h in one of the following solutions: i) a diluted extracts (ranging from 0.25 to 8.0 mg/L) containing 1% (v/v) ethanol, ii) diluted linalool (ranging from 0.1 to 3.2 mg/L) containing 1% (v/v) ethanol, iii) extract-free dechlorinated tap water containing 1% (v/v) ethanol (negative control group), and iv) extract-free dechlorinated tap water containing 0.5% (w/v) niclosamide (positive control group). The death was confirmed by the absence of snail heart beats for 2 min under Olympus microscope and the sum of the dead was used to estimate the lethal concentration (LC)_50_ and LC_90_ values for the periods of 24 h, 48 h and 96 h exposure to the leaf extract or linalool. All experiments were repeated three times under the same experimental conditions.

### Light and transmission electron microscopy study

For light microscopy, *O. hupensis* were fixed in 10% phosphate-buffered formalin solution and processed in paraffin blocks. Serial sections (5-μm thick) of the gills, salivary gland, stomach and hepatopancreas were cut, mounted on glass slides and stained with hematoxylin-eosin. They were observed using a compound light microscope (Olympus, Japan). Digital images were captured with the affiliated software of the microscope.

For electron microscopy, hepatopancreas was dissected out from three linalool treated and untreated snails, respectively and the separated samples were fixed in 2.45% glutaraldehyde and 2.45% paraformaldehyde in 0.1 M sodium cacodylate buffer (pH 7.4) at room temperature for 5 h and then at 4°C overnight. The tissues were washed in 0.1 M sodium cacodylate buffer (pH 7.4) at room temperature for 3 h and postfixed in 2% OsO_4_ at room temperature for 2 h. The specimens were washed in 0.1 M sodium cacodylate buffer (pH 7.4) at room temperature for 3 h and dehydrated with graded ethanol series and embedded in TAAB epoxy resin (Agar Scientific Ltd.). Semithin sections (80 nm) were mounted onto Formvar-coated slot grids, then contrasted with uranyl acetate and lead citrate, and examined under a Zeiss EM 902 transmission electron microscope. Semithin sections (90 nm) from the staining with uranyl acetate and lead citrate was obtained using an ultramicrotome (PowerTomeXL, RMC, Tucson, USA). Specimens were examined under a transmission electron microscopy (H-7650, Hitachi, Tokyo, Japan).

### Cercaria shedding assay

Activity assay measured on cercaria release was conducted at room temperature with a 9-h light period to determine the effect of linalool on the ability of cercaria releasing from infected *O. hupensis* by the number of the shedding cercariae within 6 hours and the time for the first cercarial release from the snails. The snails were transferred from the aquaria into Corning Costar® 6-well cell culture microplates (one snail/cell) and maintained in a diluted linalool containing 1% (v/v) ethanol or dechlorinated tap water containing the same concentration of ethanol (negative control). The microplates were observed under microscopes (Olympus, Japan) at 10-minute intervals until the released sercariae were found and the time was recorded. Six hours after the first cercarial release, the snails and cercariae were sacrificed with an incubation of the culture microplates at 80°C for 15 min. The sum of the dead cercariae at the bottom of the microplates were counted under microscopes (Olympus, Japan).

### Cercaricidal test

The cercariae of *S. japonicum* were procured from experimentally infected *O. hupensis* at room temperature and 100 ± 2 larvae were selected and exposed to the diluted linalool with dechlorinated tap water containing 1% (v/v) ethanol or dechlorinated tap water containing 1% (v/v) ethanol for 6 hours in 6-well cell culture microplates, and number of the dead cercariae was counted at 30-minute intervals. Sinking down of cercariae without movement within one minute give indication of death of the larvae, and to estimate LC_50_ and LC_90_ of linalool exposure for 30 min, 120 min and 360 min. The morphological characteristics of worms were observed under light microscope after different treatments as described above.

### Detection of tegument integrity by fluorescent probe

The effect of linalool on integrity of the cercarial tegument was evaluated using detection of tegumentary protein SjCa8, a cercariae specific molecule, which has been described by Lv et al. [19]. Briefly, after treatment with different compound for 30 min, and subsequent three washes (5 min each) with 1 × PBS, cercariae were incubated for 45 min at 37°C in PBS containing 5% bovine serum albumin (BSA, Sigma). The worms were treated for 45 min at 37°C with the primary antibodies (1:500 dilution) to recombinant SjCa8 prepared in our laboratory [19]. After an additional washing step, parasites were incubated for 1 h at room temperature with Cy3-conjugated anti-mouse IgG diluted at 1:100, washed thrice and then observed under an FV 500-IX81 laser scanning confocal system (Olympus, Japan) with Krypton/Argon laser and appropriate filters.

### Skin penetration and tissue migration assay

Twenty four Balb/C mice were randomly allocated to three groups with eight mice in each group. All mice were infected percutaneously with 30 *S. japonicum* cercariae. Prior to the infection of animals, the cercariae were exposed for 15 min to 0.1 mg/L, 1 mg/L linalool, or dechlorinated tap water containing 1% (v/v) ethanol, respectively. The mice were sacrificed 30 min after the challenge and the shaved skins exposed to the larvae were cut into pieces and then cultured for 24 h at 37°C, 5% CO_2_ in 6-well cell culture microplates in RPMI 1640 medium supplemented with antibiotics (100 U/ml of penicillin and 100 μg/ml of streptomycin) and 10% fetal calf serum (Invitrogen, USA) in quintuplicate. The penetrated larvae were collected and counted to evaluate the linalool's effect on skin penetration and migration by cercariae.

### Worm recovery and tissue sampling

Twenty four Balb/C mice (8 mice/group) were challenged with cercariae after treatment as the protocol of "Skin penetration and tissue migration assay". Forty-five days after the challenge infection, the adult worm recovery and tissue sampling were performed according to our previously described study [19]. In brief, all mice were sacrificed by cervical dislocation and adult worms were recovered from hepatic portal system and mesenteric veins. The percentage of worm reduction for each group was calculated according to the following formula: % reduction = (1-mean number of worms in experimental mice/mean number of worms in challenge control mice) × 100 [20]. The livers and intestines of mice from all groups were collected and weighed, and a known portion (0.5 g) of the intestine and liver from each animal were fixed and serial sections was prepared as description above for routine histopathological examination of granuloma in the organs. Moreover, same part of livers and intestines were completely digested with potassium hydroxide (4%) overnight at 37°C on a rocking platform. The eggs in the digest were put on the glass slide and counted under light microscopy. The calculation of egg reduction rate for each gram of liver or intestine was based on the following formula: % reduction = [(average number of eggs/g liver or intestine in control group - average number of eggs/g liver or intestine in experimental group)/average number of eggs/g liver or intestine in control group] × 100 [21]. Egg reduction rate for each adult female was calculated as following formula: egg reduction rate for each adult female (%) = [(average number of eggs/g liver or intestine/number of female in control group - average number of eggs/g liver or intestine/number of female in experimental group)/average number of eggs/g liver or intestine/number of female in control group] × 100.

### Statistical analysis

Data were expressed as the mean ± standard deviation (SD) and *P < 0.05, determined by Student’s t test (GraphPad Prism 5), was considered significant. LC_50_, LC_90_ and their respective 95% confidence limit (95% CL) were obtained by nonlinear regression using the SPSS program version 16.0.

## Results

### SC-CO_2_ extraction and GC-MS analysis

The composition of the *C. camphora* leaf extracts obtained by means of supercritical CO_2_. Table 1 gave 44 compounds of the supercritical extracts identified from the supercritical extracts by GC-MS, representing 94.37% the total extract. Four major constituents with concentrations higher than 5% as the percentage peak area were linalool (43.73%), camphora (14.43%), 1,8-cineole (10.46%) and safrole (7.08%) (Table 1).

**Table 1:** **Components in the leaf extract of** ***C. camphora*** **with their molecular weight, molecular formula and relative content**

Peak no.	Component of volatile oil	Molecular weight	Molecular formula	Relative content (%)
1	Acetone	58	C_3_H_6_O	0.359
2	2-pentene	70	C_5_H_10_	0.129
3	2,4-hexadiene	82	C_6_H_10_	0.038
4	Cyclopentene,1-methy1-	82	C_6_H_10_	0.032
5	1-penten-3-ol,3-menthyl	100	C_6_H_12_O	0.010
6	4-pentanedione	100	C_5_H_8_O_2_	0.045
7	3-penten-2-one,4-methyl	98	C_6_H_10_O	0.152
8	3-hexen-1-ol	100	C_6_H_12_O	0.089
9	α-thujene	136	C_10_H_16_	0.214
10	4,4-dimethyl-2-pentynal	100	C_7_H_10_O	0.015
11	Camphene	138	C_10_H_16_	0.265
12	1,3,8-para-menthatriene	134	C_10_H_14_	0.099
13	α-terpinene	136	C_10_H_16_	0.212
14	p-cymene	134	C_10_H_14_	0.237
15	dl-limonene	136	C_10_H_16_	0.281
16	1,8-cineole	154	C_10_H_18_O	10.457
17	Cis-ocimene	136	C_10_H_16_	0.318
18	Trans-ocimene	136	C_10_H_16_	0.215
19	γ-terpinene	136	C_10_H_16_	0.012
20	Cis-linalooloxide	170	C_10_H_18_O_2_	0.115
21	Trans-linalooloxide	170	C_10_H_18_O_2_	0.148
22	Linalool	154	C_10_H_18_O	43.732
23	α-pinene	136	C_10_H_16_	1.111
24	β-phellandrene	136	C_10_H_16_	2.231
25	Alloocimene	136	C_10_H_16_	0.168
26	4-methyl-3-decen-2-ol	170	C_11_H_22_O	0.162
27	Camphora	152	C_10_H_16_O	14.431
28	Epoxylinalol	170	C_10_H_18_O_2_	0.802
29	Borneol	154	C_10_H_18_O	0.985
30	linalool, Z-pyranic acid	170	C_10_H_18_O_2_	0.058
31	4-terpineol	154	C_10_H_18_O	1.333
32	3-hexenyl butyrate	170	C_10_H_18_O_2_	0.183
33	α-terpineol	154	C_10_H_18_O	2.57
34	Nerol	154	C_10_H_18_O	0.474
35	Trans-sabinene hydrate	154	C_10_H_18_O	0.13
36	Trans-geraniol	154	C_10_H_18_O	0.77
36	α-citral	154	C_10_H_18_O	0.298
38	Safrole	162	C_10_H_10_O_2_	7.079
39	Trans-aryophyllene	204	C_15_H_24_	0.29
40	α-umulene	204	C_15_H_24_	0.678
41	Spathulenol	220	C_15_H_24_O	1.814
42	Cis-α-santalol	220	C_15_H_24_O	0.597
43	Nerolidol	222	C_15_H_26_O	0.811
44	Soeugenol	164	C_10_H_12_O_2_	0.579

### Molluscicidal effects against *O. hupensis*

**Figure 1:**
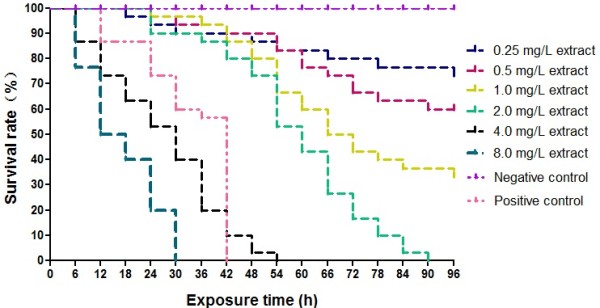
**Molluscicidal activity of** ***C. camphora*** **leaf extracts against** ***O. hupensis*** **in different time intervals and concentrations in the laboratory.**

**Figure 2:**
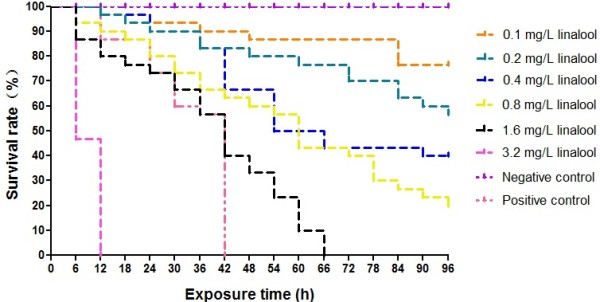
**Molluscicidal activity of linalool from** ***C. camphora*** **leaf extracts against** ***O. hupensis*** **.**

Molluscicidal activities of *C. camphora* leaf extracts freshly prepared at concentrations ranging from 0.25 to 8.0 mg/L and linalool (0.1, 0.2, 0.4, 0.8, 1.6, 3.2 mg/L) on *O. hupensis* snails were determined by the bioassay. The groups treated with the leaf extracts or linalool exhibited an exposure concentration- and time-dependent mortality against the intermediate host snails (Figures 1 and 2). The LC_50_ for the supercritical extracts and linalool after 96 h exposure were given in Table 2. Taken into the consideration of the percentage of linalool (43.72%, see Table 1) in the extracts, the molluscicidal activity of linalool (LC_50_ and LC_90_ at 96 h: 0.25 mg/L and 1.00 mg/L, respectively) was close to that of the leaf extracts (LC_50_ and LC_90_ at 96 h: 0.63 mg/L and 2.51 mg/L, respectively), suggesting that linalool might be the major component of molluscicidal activities in *C. camphora* leaf extracts.

**Table 2:** **Molluscicidal activity of** ***C. camphora*** **leaf extracts and linalool against** ***O. hupensis*** **after 96 h exposure (mg/L)**

	***C. camphora***leaf extracts	Linalool
LC_50_ (95% CL)	0.63 (0.47-0.81)	0.25 (0.19-0.32)
LC_90_ (95% CL)	2.51 (1.81-4.18)	1.00 (0.72-1.67)

### Light microscope and TEM study of snails treated with linalool

**Figure 3:**
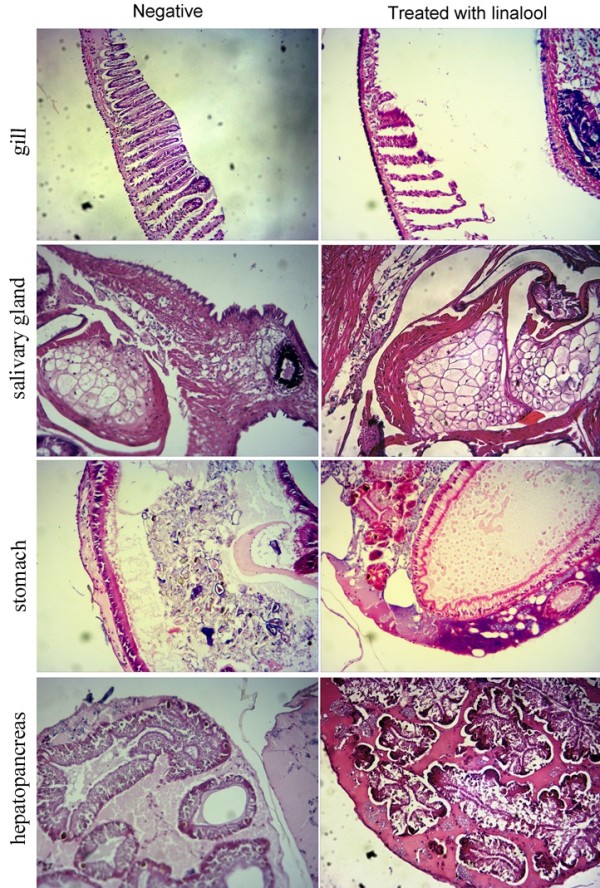
**Typical light micrograph of gills, salivary glands, stomach and hepatopancreas of** ***O. hupensis*** **treated for 1 h with 1.0 mg/L linalool (linalool-treated group) or without linalool (negative group).**

**Figure 4:**
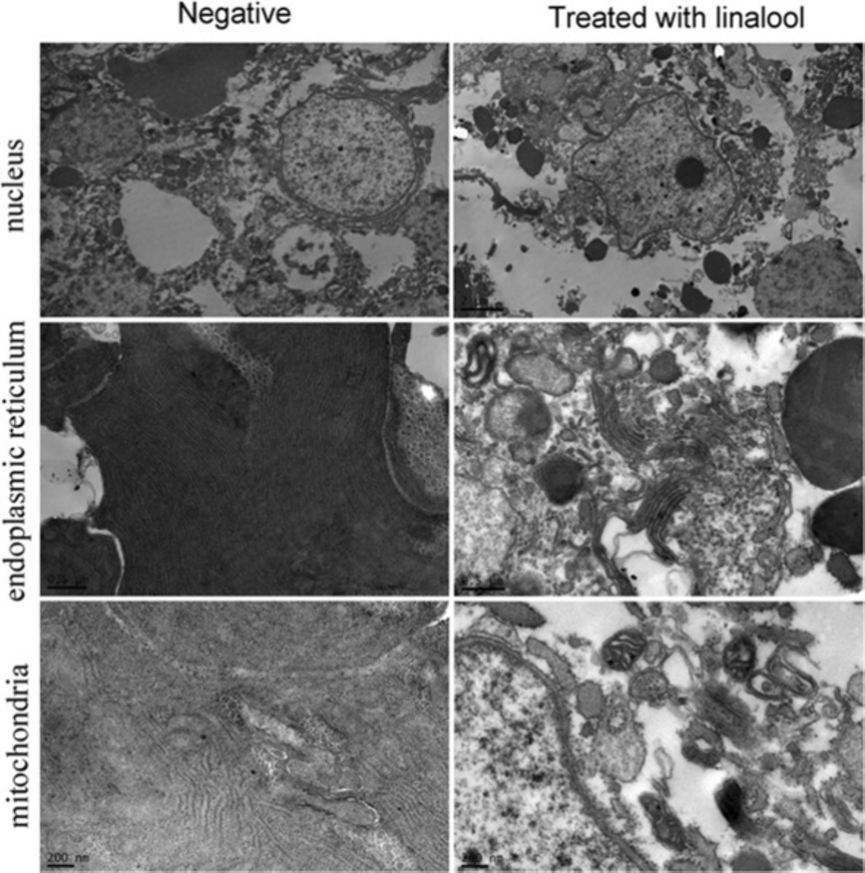
**Representative Transmission electron micrograph of nuclei, rough endoplasmic reticulum and mitochondria in hepatopancreas of** ***O. hupensis*** **treated 1 h with 1.0 mg/L linalool (linalool-treated group) or without linalool (negative group).**

The histological sections examined disclosed that both the numbers and shape of the salivary gland cells in experimental groups and negative group were similar, and stomach in all groups were characterized by the integrated membrane and the aligned columnar epithelium on the basal membrane (Figure 3), demonstrating a lack of effect of linalool on the digestive system of *O. hupensis*. Hematoxylin-eosin staining revealed that in the control snails, most of the epithelial lining of the gills consisted of columnar cells with long cilia (Figure 3), however, in the linalool-treated snails, the gills exhibited an obvious loss of cilia and high degeneration of columnar cells (Figure 3). The combined hepatic and pancreatic tissue are collectively called the digestive gland or hepatopancreas. The hepatopancreas of linalool-treated snails shrank and separated from the connective parenchyma, and characterized by much smaller lumen tubules and less oval dark granules as compared to the negative group (Figure 3). The results indicated that damages to gills and hepatopancreas might be the major causes of death in the snails after exposure to linalool.Transmission electron microscope showed that in hepatopancreas cells of the untreated snails, the chromatin evenly distributed in the oval nucleus, while in snails treated with 1.0 mg/L linalool, the irregular shape cell nucleus appeared an obvious chromatin pyknosis (Figure 4). Meanwhile, in the cells from experiment groups, the rough endoplasmic reticulum was apparently swollen, and significant fragmentation was evident, but characteristic stacks of lamellar endoplasmic reticulum were seldom found. And TEM revealed the presence of turgescence and vacuolization in mitochondria, as compared to the normal snails (Figure 4).

### Cercaria shedding assay

**Figure 5:**
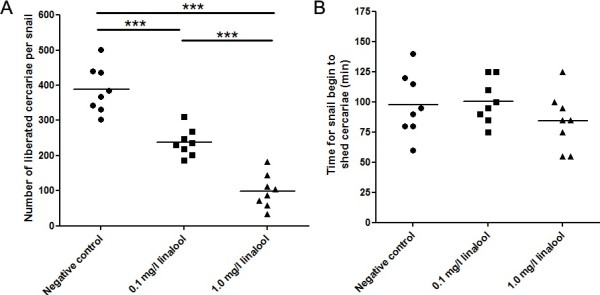
**Numbers of liberated cercariae from** ***O. hupensis*** **exposed to different compounds were counts (A) and the time for the cercariae liberates were recorded (B) to evaluate the effect of linalool on cercariae shedding from the snails.**

Data concerning the number of cercariae shed from 24 infected *O. hupensis* (8 snails per groups) were counted, respectively, and given in Figure 5A, which covered a 9-h light period. When the infected snails were exposed to 0.1 mg/L or 1.0 mg/L linalool for 30 min, they liberated significantly fewer larvae (237.30 ± 13.82 and 99.38 ± 16.92, respectively) than the unexposed snails (number of the shedded cercariae: 388.30 ± 23.54, *P* < 0.001, Figure 5A).

The time of the first cercarial release from each infected snail grouped as above was recorded individually and the difference between the groups was not statistically significant (*P* > 0.05, Figure 5B).

### Cercaricidal test

**Figure 6:**
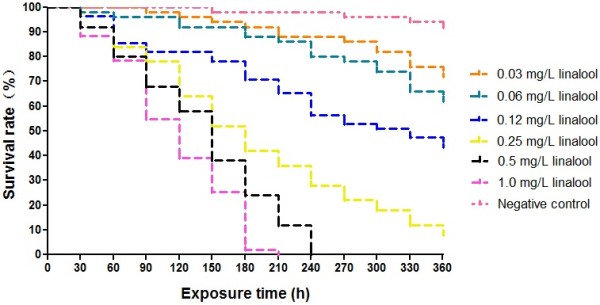
**Cercaricidal activity of linalool against** ***Schistosoma japonicum*** **.**

**Figure 7:**
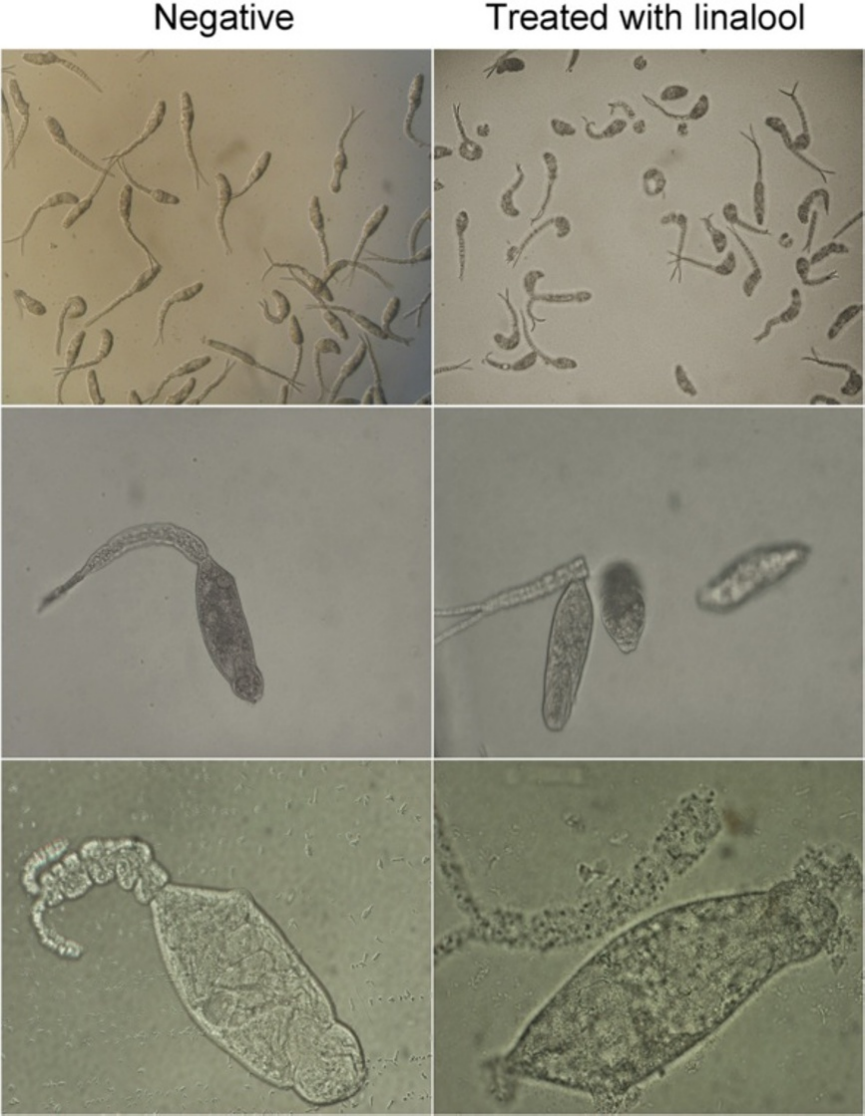
**Morphological changes of cercariae with exposure to linalool were observed under the light microscope (upper row, ×5; middle row, ×40; lower row, ×100).**

**Figure 8:**
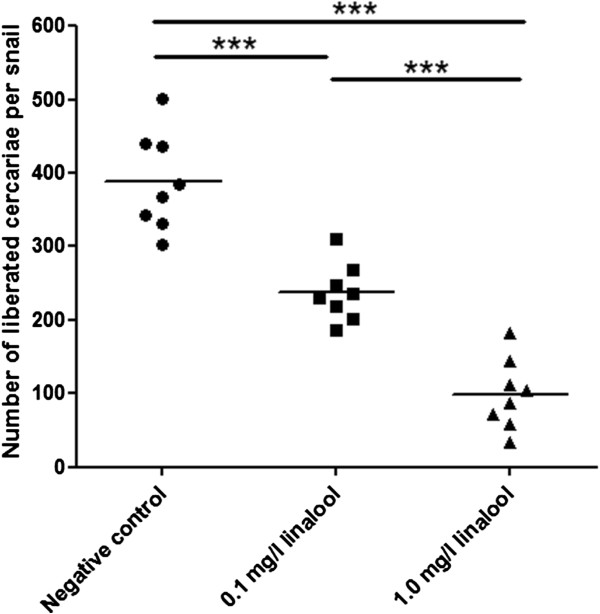
**Recovery of schistosomula from mouse skin challenged with untreated or linalool-pretreated cercariae.**

The larvicidal properties of linalool were directly determined against *S. japonicum* and exhibited a time- and concentration-dependent pattern (Figure 6). The concentrations needed to kill 90% cercariae (LC_90_) within 360 min and all cercariae within 150 min of exposure were 0.25 mg/L and 1.0 mg/L, respectively (Figure 6, Table 3). The results showed that linalool not only was harmful to snails, but also exhibited promising killing effects on the cercariae, which was reflected in sharply fewer cercariae produced and liberated in the linalool-treated groups as compared to the control group.Though no significant mortality could be observed 30 min after exposure to different concentration of linalool (Figure 6), increasing numbers of larvae in experimental group were not intact. As can be seen in Figure 7, significantly more tailless worms were visualized in pre-treated group with 0.1 mg/L linalool for 30 min, compared to untreated groups, indicating an evident acceleration in cercarial decaudation by linalool. Remarkably, the detached tails and the head-tail junctions of cercaria after the treatment of linalool appeared visible breakages (Figure 8).

**Table 3:** **Cercaricidal activity of 6 h exposure to linalool (mg/L)**

	Linalool
LC_50_ (95% CL)	0.07 (0.06-0.08)
LC_90_ (95% CL)	0.25 (0.19-0.35)

### Detection of tegument integrity

To determine whether linalool exhibited damage to integrity of cercariael tegument, we used immunofluorescence to localize a cercaria-specific tegumental protein SjCa8 as described above. Thirty minutes after treatment with linalool (0.1 mg/L), a majority of living cercariae began to shed their tails and the detached tails started to grow a large number small bulbous structures throughout whole tail region. Moreover, fluorescence-labeled particles were leaked from the head-tail junctions of the linalool-treated larvae and visualized under a fluorescence microscope in comparison to untreated parasites (Figure 7).

### Skin penetration assay

**Figure 9:**
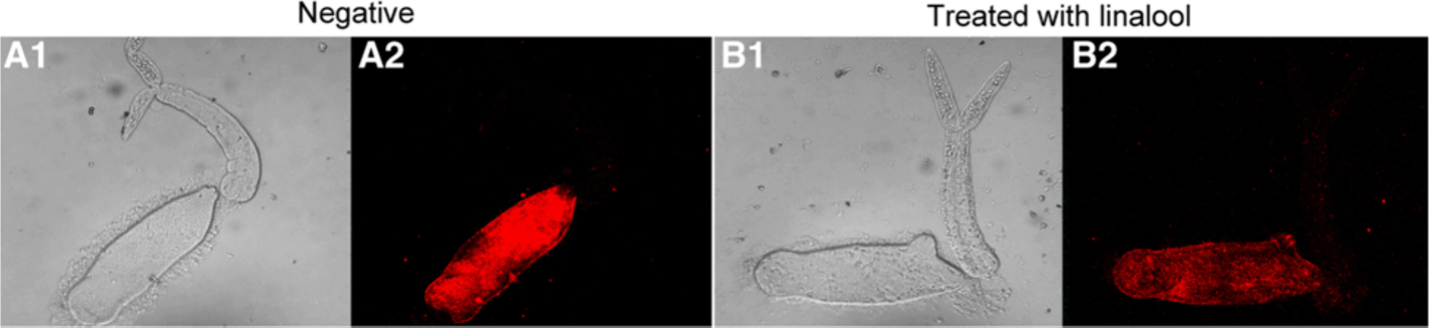
**Morphological changes of tegument of cercariae exposed to linalool were observed under the fluorescence microscope (×40). A1** and **B1** are phase-contrast fields of the fluorescent fields **A2** and **B2**, respectively.

After an incubation in RPMI 1640 medium for 24 h, the numbers of larvae recovered from mouse skin challenged with 30 cercariae, which were exposed to different compounds, were counted under a microscope. Figure 9 showed that the recovered schistosomulum in experimental groups treated with 0.1 mg/L (12.2 ± 2.5) or 1.0 mg/L linalool (11.8 ± 4.3) appeared significantly less than in the negative control (21.6 ± 3.7) (*P* < 0.001), and the number of shistosomulum in 0.1 mg/L linalool-treated group achieved the similar level as the group treated with 1.0 mg/L linalool (*P* > 0.05).

### Protective efficacy against challenge infection

Administration of 0.1 mg/L and 1.0 mg/L linalool induced highly significant worm burden reduction of 27.42% and 60.75% (Table 4), respectively, in agreement with highly significant decreases in worm egg counts in mouse liver (25.03% and 58.12% reduction, respectively) and intestine (23.36% and 64.90% reduction, respectively). However, the number of egg counts per female worm recovered from tissues were equivalent between the control group and treatment groups (*P* > 0.05, Table 4).

**Table 4:** **Treatment and protection**

Groups	Female	Male	Total	Reduction	Egg number/g liver	Reduction	Egg number/g liver	Egg number/g intestine	Reduction	Egg number/g intestine
	Worms	Worms	Worms	(%)	(×10^5^)	(%)	/female worm (×10^4^)	(×10^5^)	(%)	/female worm (×10^4^)
Control	11.50 ± 1.93	11.50 ± 1.60	23.25 ± 3.24		4.73 ± 1.02		4.14 ± 0.81	5.71 ± 0.98		5.01 ± 0.82
0.1 mg/l linalool	8.38 ± 1.92	8.5 ± 1.69	16.88 ± 3.48	27.42^**^	3.54 ± 0.90	25.03^*^	4.25 ± 0.62	4.37 ± 0.60	23.36^**^	5.39 ± 0.97
1.0 mg/l linalool	4.50 ± 1.60	4.63 ± 1.41	9.13 ± 3.00	60.75^***^	1.98 ± 0.66	58.12^***^	4.50 ± 0.91	2.00 ± 0.76	64.90^***^	4.47 ± 0.75

Burdens and reduction rates of worm, hepatic egg, and intestine egg were calculated as described in Section "Methods" *P < 0.05, **P < 0.01 and ***P < 0.001, calculated by Student’s t-test.

### Hepatic and intestine egg reduction

**Figure 10:**
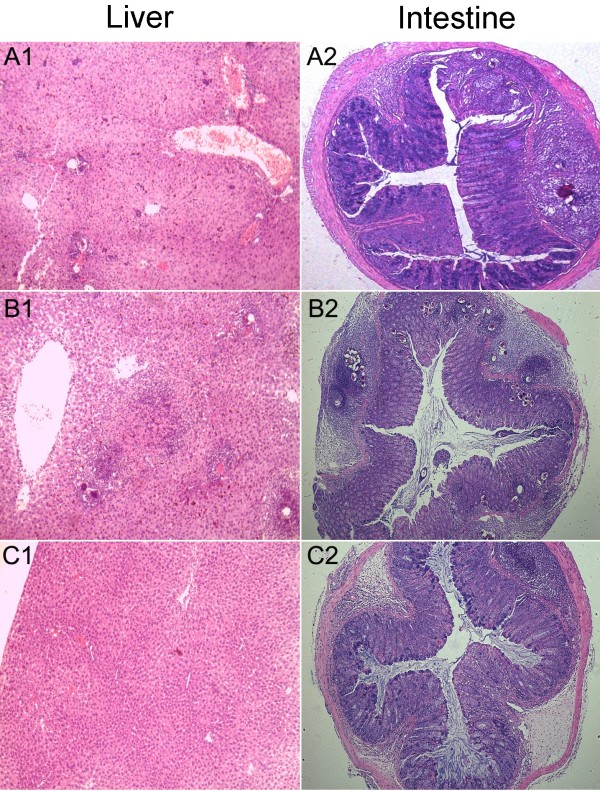
**Photomicrographs (×40) of liver pathology of the mice 45 days after challenge infection. A1** and **A2**: mice were challenged with linalool-pretreated cercariae; **B1** and **B2**: mice were infected with untreated cercariae; **C1** and **C2**: normal mice without challenge infection.

Histopathological examination showed the presence of granulomata in the livers and the intestinal submucosa of all animals infected with cercariae (Figure 10). Accompanied with the lower number of recovered worms and egg counts in host tissues, mice challenged with linalool-pretreated cercariae also displayed a marked decrease in the number and size of egg-induced granulomas, surrounded by variable number of inflammatory cells, in the tissues compared with untreated group (Figure 10).

## Discussion

Parasitic diseases, including malaria, schistosomiasis, filariasis and Chagas disease, remain a major public health threat affecting more than 30% of the human population in the world. Unfortunately, parasite infection can hardly be prevented by vaccination in most instance and many parasites have become resistant to the available pharmaceutical medicines. There is an increasing awareness of the potential of natural products from plants, which may lead to the development of much-needed new anti-parasitic drugs, with interesting biological activities of cytotoxic and anti-parasitic properties [22–25]. *Cinnamomum* extracts have been identified as preferred natural antiparasitic agents for the treatment of ectoparasitic louse [26] and mite [27], entoparasites of *Dactylogyrus intermedius* (a parasitic helminth) [28], *Tetratrichomonas gallinarum* and *Histomonas meleagridis* (protozoan parasites) [29], and malaria vector of *Anopheles gambiae* [30]. No systematic evaluation of molluscicidal or cercaricidal activity of *C. camphora* extracts against *O. hupensis* or *S. japonicium* has been carried out. In this study, we characterized the promising molluscicidal effect of *C. camphora* against schistosomiasis vector snail (*O. hupensis)* and identified the chemical composition of exacts of *C. camphora* leaves by GC-MS analysis. Taken into consideration of the highest abundance of *C. camphora* leaf extracts and known molluscicidal activities against schistosomiasis and fascioliasis vector snails of *Biomphalaria alexandrina*, *Bulinus truncatus* and *Lymnneae natalensis* [31], linalool investigated in the present report exhibited a highly significant molluscicidal effect on *O. hupensis*, the unique intermediate host of *S. japonicum*, as compared to control. The most evident histopathological alterations occurred in the hepatopancreas, the main metabolic and detoxification organ in molluscs [32], and the gills, which are involved in the transport of respiratory gases and regulation of ionic and osmotic balances in snails [33]. Severe damage to these two organs, therefore, likely lead to a disruption of the osmoregulation and a reduction of oxygen consumption, which are critical to snail survival [34, 35].

Interestingly, linalool also displayed a considerable cercaricidal activity against *S. japonicium* accompanied with an inhibition of larvae shedding from snails and an obvious damage to tegumental integrity of cercariae exposed to the compound. The tegument of cercariae is a thin syncytial layer that covers the whole worm, which constitutes the major host-parasite interface after transformation of schistosomula and serves as a barrier preventing the loss of fluid, proteins and ions from breakage between body-tail joint and maintaining osmotic balance during penetration into and migration in the skin of hosts [36, 37]. Hence, the disruption of cercarial tegument by exposure to linalool will impair the crucial functions of larval tegument in immune evasion and parasite survival, which was coincident with the results of a significant reduction of recovered larvae in skin penetration assay and adult worm burden in animal challenge experiment. Nevertheless, linalool exhibited inapparent ovicidal properties on *S. japonicum* according to the similar egg counts in liver or intestine per female adult in all infected animals in this study.

Linalool, a monoterpene alcohol, is the most abundant component of *C. camphora* leaf extracts tested by GC-MS in this study and the major constituents of *Aniba rosaeodora*, *Arisaema franchetianum*, *Arisaema lobatum*, *Croton cajucara* and *Ocimum forskolei* essential oils [38–41]. Linalool-rich extracts from various plants present properties against bacteria [42], viruses [43], insects [44] and parasites (protozoa and helminths) [45–47, 40] and the results presented in this work further support the molluscicidal and anti-parasite activity of linalool-rich exacts from plants. The extreme molluscicidal and cercaricidal properties of linalool offer a chance to develop a new lead compound for novel anti-schistosomal drugs.

## Conclusion

In summary, in this work we first report linalool, the most abundant components from *Cinnamomum camphora* leaf extracts and a potentially important target for development of natural and novel agents for the control of schistosomiasis. Our analysis of molluscicidal activity and cercaricidal property of linalool against *Oncomelania hupensis* and *Schistosoma japonicium*, respectively, provide insights into antiparasitic treatment of this eco-friendly compound. Perhaps most striking are the characteristics of linalool that apparently causes damages to gills and hepatopancreas of the snails, and disruption of cercarial tegument. The results reported here should catalyze future studies of linalool and development of an alternative anti-schistosomal drug.

## Authors’ information

Yang Fan, Long Erping, Wen Juhua and Cao Lei are joint first authors.
